# COVID‐19 vulnerability among people who use drugs: recommendations for global public health programmes and policies

**DOI:** 10.1002/jia2.25551

**Published:** 2020-07-08

**Authors:** Ian W Holloway, Anne C Spaulding, Ayako Miyashita Ochoa, Laura A Randall, Adrian R King, The HBOU Study Team, Paula M Frew

**Affiliations:** ^1^ Department of Social Welfare UCLA Luskin School of Public Affairs Los Angeles CA USA; ^2^ Department of Epidemiology Emory University Atlanta GA USA; ^3^ UNLV School of Public Health and UNLV Population Health & Health Equity Initiative University of Nevada Las Vegas Las Vegas NV USA

**Keywords:** COVID‐19, substance use, opioid crisis, homelessness, social determinants

As cases of COVID‐19, the disease caused by the novel coronavirus (SARS‐CoV‐2), continue to spread globally, public health experts have warned about the devastating impact this pandemic may have on society’s most vulnerable [1]. Meanwhile, another public health crisis, the opioid epidemic, rages on throughout the United States [2, 3]. In other parts of the world, the use of opiates and opioids has remained relatively stable; however, in Eastern Europe, Russia and Central Asia opiate use has increased [4]. In these regions, the prevalence of injection drug use is high, as is prevalence of HIV among people who inject drugs – over 40% in major Russian cities according to recent estimates [5]. Worldwide nearly half a million people died as a result of drug misuse in 2015; 168,000 of those deaths were due to overdose [6]. People living with HIV (PLWH) face higher risk of opioid misuse than HIV‐negative people, in part because PLWH are more likely to suffer chronic pain and receive opioid analgesic treatment for symptom relief [7, 8].

People who use drugs (PWUD) face unique vulnerabilities in the COVID‐19 era, including elevated homelessness and frequent interaction with criminal justice systems [9]. In the United States alone, approximately four million people experiencing homelessness cycle in and out of a variety of high‐risk settings, including street encampments, shelters, jails and prisons. Each of these contexts present significant challenges to limiting the spread of COVID‐19. On the streets, for example, the very supplies needed to follow World Health Organization (WHO) recommended hand hygiene guidelines [10], including access to clean water, soap and hand sanitizer, are scarce. Prison overcrowding, which was an international health concern prior COVID‐19 [11], has reached a crisis point. Limited COVID‐19 testing in correctional settings, scarce PPE for inmates and staff and COVID‐19 outbreaks in prisons across the world make incarcerated PWUD especially vulnerable for COVID‐19 infection and resulting negative health outcomes [12].

For PWUD in community settings, disruptions in global supply chains and implementation of physical distancing measures in response to the COVID‐19 pandemic may limit access to drugs and harm reduction services. The popular press has documented limited availability of fentanyl and other synthetic opioids due to closings of chemical manufacturing plants in Wuhan, China [13]. Rising nationalism evidenced by policies banning pharmaceutical and PPE exports, such as those instituted by the United States and India [14], and border closings, such as those implemented in South America [15], may decrease the availability of certain drugs, including synthetic opioids. Whether disruptions in the drug supply chain are real or imagined, the fear experienced by PWUD has an immeasurable effect on their wellbeing. In addition, harm reduction programmes, including naloxone distribution and syringe exchange, have become increasingly difficult to access under “shelter in place” restrictions [16].

So what can community advocates, researchers and international stakeholders do to address COVID‐19 vulnerability among PWUD? A comprehensive policy response must be multi‐pronged and dedicated to improving public health programmes that serve PWUD and the communities in which they live. Investment in public health infrastructure needs to incorporate investment in addressing social determinants of health, including homelessness. In France, temporary homeless shelters have opened across the country to accommodate those unable to shelter in place [17]. A recent analysis estimated that 400,000 new beds are needed to meet the emergency accommodation and social distancing needs of the single adult homeless population in the United States on a given day during the COVID‐19 pandemic [18]. Investment in temporary housing needs is paramount for effectively addressing COVID‐19 among PWUD.

Decriminalization of PWUD and access to harm reduction services for PWUD must also be prioritized during the COVID‐19 pandemic. Scholars have called for police and courts to immediately suspend arresting and sentencing people for low‐level crimes and misdemeanors [19], which often include possession of drugs. Over 70 countries worldwide have introduced syringe exchange programmes [20] and naloxone has saved tens of thousands of lives in the United States alone [21]; however, access to these harm reduction services remains difficult in many countries. Anticipated scarcity of syringes and other injection equipment during the COVID‐19 pandemic may prompt PWUD to share syringes or experiment with new drugs or new drug use habits. To prevent outbreaks of HIV, HCV and other infectious diseases among PWUD, policy makers in nations with high rates of opioid use, including Russia and other Eastern European and Central Asian countries where new COVID‐19 cases are beginning to surge [22], would be wise to consider implementing government‐supported programmes to purchase and allocate syringes. WHO‐endorsed naloxone distribution and training programmes should be re‐considered in contexts where harm reduction programmes are outlawed [6].

Finally, because various physical distancing measures in response to COVID‐19 are anticipated to remain in place for the foreseeable future, PWUD may have difficulty accessing healthcare services, including opioid substitution therapy, medication‐assisted treatment and other substance use treatment services. Telemedicine has been successfully implemented during other global emergencies [23] and presents a key opportunity for helping PWUD stay connected to services while protecting themselves against COVID‐19 infection. PWUD in greatest need will require sustained access to technology that allows them to leverage telemedicine to facilitate engagement with critical healthcare services, including continuous contact with counsellors and addiction medicine specialists. In Canada, opioid substitution therapy is prescribed and administered through daily dispensing at pharmacies or as a take‐home medication [24]. During the COVID‐19 pandemic some governments have recommended home delivery of HIV medications [25]; similar considerations for opioid substitution therapy and medication‐assisted treatment may be possible in certain contexts. Uninterrupted access to cellular and internet service will also be crucial to the success of these programmes.

As highlighted here, PWUD are especially vulnerable to COVID‐19 given their increased risk for homelessness, interactions with criminal justice systems and need to access in‐person services for substance use treatment. International leaders should consider programmes and policies outlined here in order to avoid a more concentrated epidemic of COVID‐19 among PWUD. Millions of lives across the globe depend on it.

## COMPETING INTERESTS

The authors declare no competing interests.

## AUTHORS’ CONTRIBUTIONS

IH and PF co‐conceptualized the Viewpoint and wrote and edited the final version. IH took the lead and AS, AM and PF supported with writing and editing. LR and AK provided additional feedback based on comments from AS, AM and PF. The HBOU Study Team consists of a cross‐university collaborative working on issues related to opioid use and health behaviours.
